# Supplementary material for: Recruitment and positioning determine the specific role of the XPF‐ERCC1 endonuclease in interstrand crosslink repair
Source: EMBO J. 2017 Mar 14;36(14):2034–46. doi: 10.15252/embj.201695223 (PMC5510004; doi:10.15252/embj.201695223)
Supplement: Supplementary file 1 — Appendix [file EMBJ-36-2034-s001.pdf]

Appendix

Table of contents:

Appendix Figures S1 and S2

Appendix figure legends

Appendix Supplementary Methods

Appendix References

Appendix Fig S1

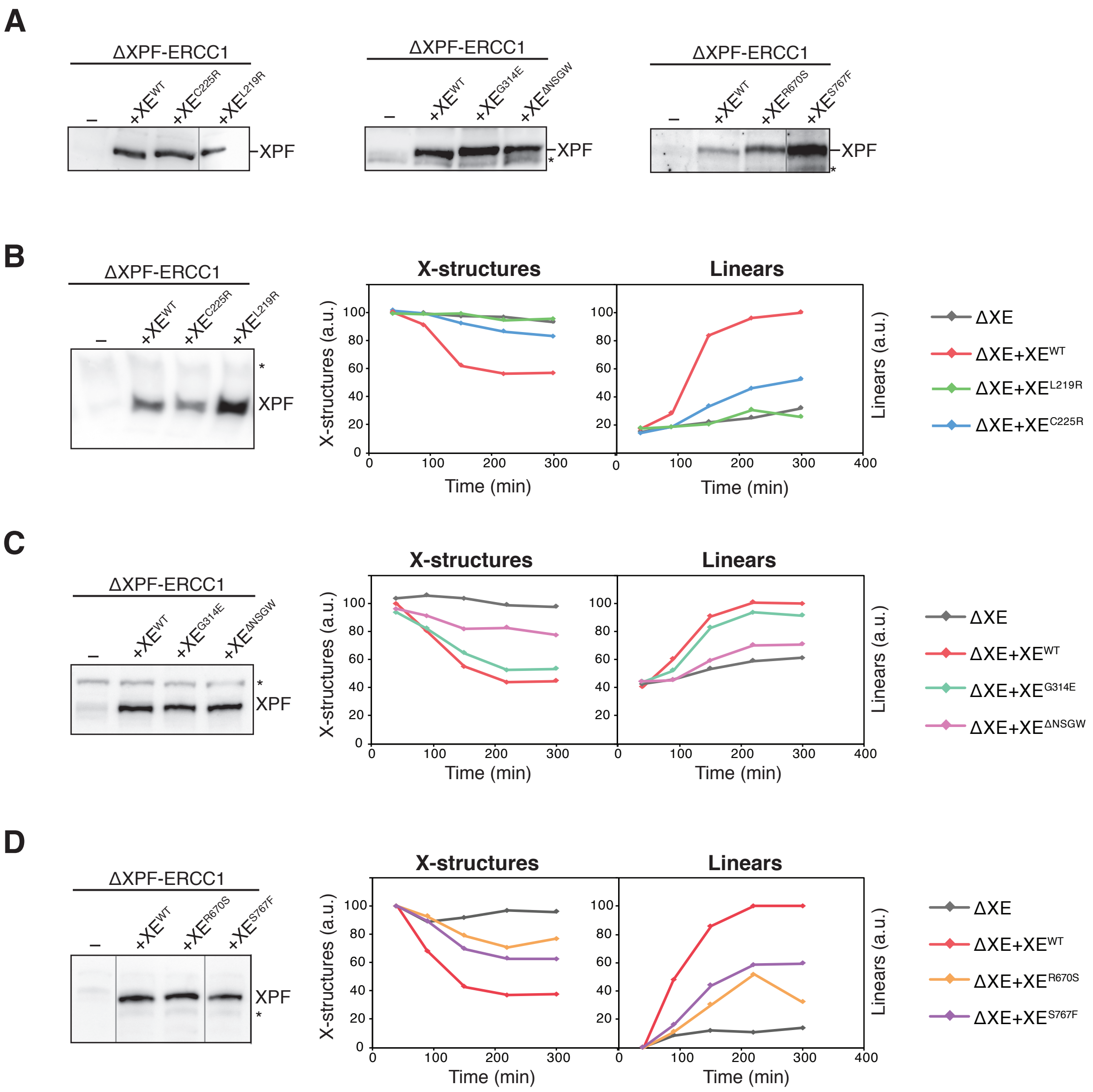

Appendix Fig S2

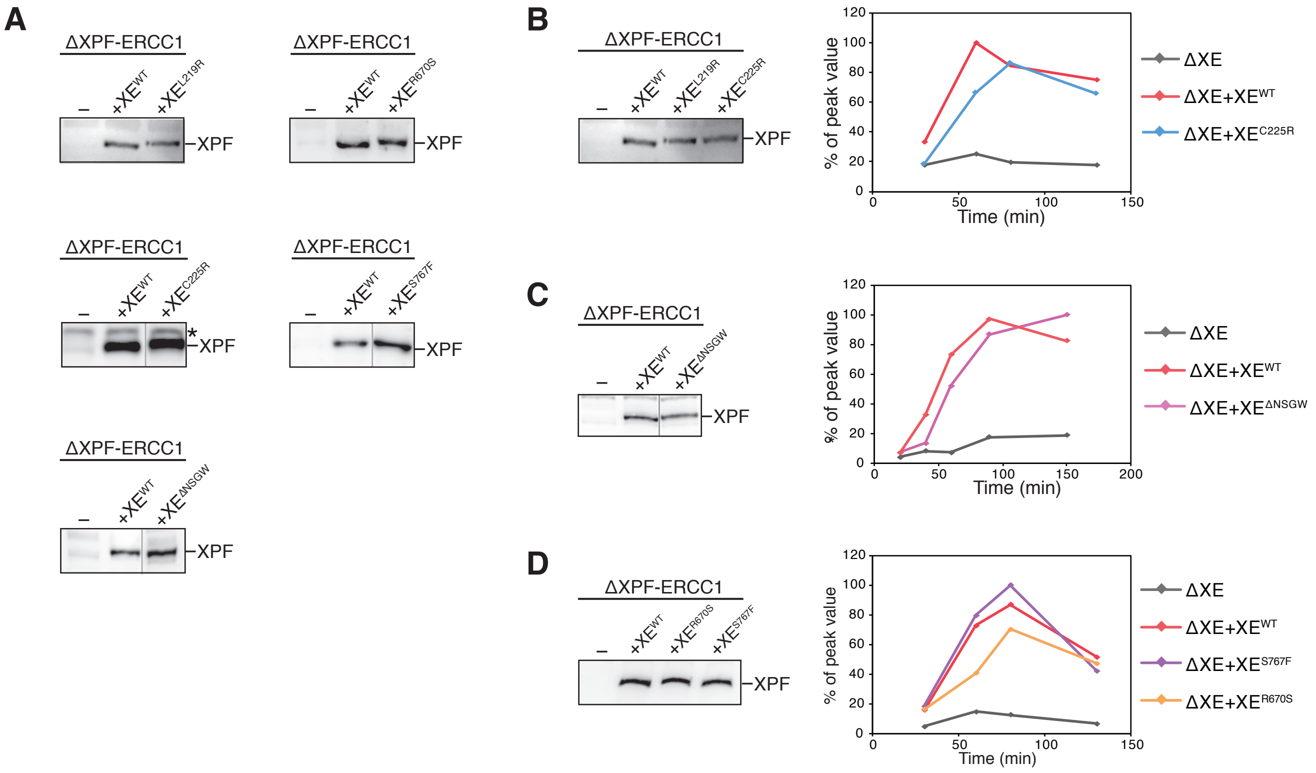

Appendix figure legends

Appendix Fig S1: Separation of function mutants are defective in ICL unhooking. **A**) XPF-ERCC1 depleted (ΔXE) NPE or XPF-ERCC1 depleted NPE supplemented with wild type (XE^{WT}) or indicated mutant XPF-ERCC1 (XE^{MUT}) were analyzed by western blot using αXPF antibodies to verify depletion and the levels of added recombinant protein. Extracts were used to perform unhooking assay depicted in Fig 4 B-D. **B**) Replicate of Fig 4B. XPF-ERCC1-depleted (ΔXE) or XPF-ERCC1-depleted egg extract supplemented with wild type (XE^{WT}) or mutant XPF-ERCC1 (XE^{MUT}) were incubated with pre-labeled pICL. Repair products were isolated at indicated times, linearized with HINCII, and visualized on a denaturing agarose gel. The X-structures and linear products were quantified and plotted (right panel). The extracts used were analyzed by western blot using α-XPF antibodies to verify depletion and the levels of added recombinant protein (left panel). **C**) and **D**) as in (B) but using different XPF-ERCC1 mutant complexes. Replicates of Fig 4C and Fig 4D. Line within blot indicates position where irrelevant lanes were removed. *, background band.

Appendix Fig S2: Recruitment of XPF-ERCC1 mutants to the ICL during repair. **A**) XPF-ERCC1 depleted (ΔXE) NPE or XPF-ERCC1 depleted NPE supplemented with wild type (XE^{WT}) or indicated mutant XPF-ERCC1 (XE^{MUT}) were analyzed by western blot using αXPF antibodies to verify depletion and the levels of added recombinant protein. Extracts were used to perform ChIP in Fig 5 B-F. **B**) Replicate of Fig 5C. pICL was replicated in XPF-ERCC1 depleted (ΔXE) or XPF-ERCC1-depleted egg extract supplemented with wild type (XE^{WT}) or mutant XPF^{C225R}-ERCC1 (XE^{C225R}). Samples were taken at various times and immunoprecipitated with α-XPF antibodies. Co-precipitated DNA was isolated and analyzed by quantitative PCR using the primers depicted in Fig 5A. The qPCR data was plotted as the percentage of peak value with the highest value within one experiment set to 100% (right panel). The extracts used were analyzed by western blot using α-XPF antibodies to verify depletion and the levels of added recombinant protein (left panel). Of note: the XPF^{C225R} was analyzed in the same experiment as depicted in Fig 5B. **C**) and **D**) as in (B) but for the indicated XPF-ERCC1 mutant complexes. Replicates of Figures 5D, E and F. Line within blot indicates position where irrelevant lanes were removed. *, background band.

Appendix Supplementary Methods

Protein expression and purification

The his-PCNA protein was purified as previously described (Kochaniak *et al.*, 2009). The *xl*SLX4 BTB domain (residues 547-685) was amplified using PCR, with an N-terminal 10xHis-tag and flanking restriction sites, and cloned into pETDuet-1. The construct was transformed into Rosetta (DE3) competent cells for IPTG-induced overexpression. 150 µL 1M IPTG was added to 400 mL culture at OD_{600}=0.6 and incubated for 20 hours at 20°C. Cells were collected by centrifugation, resuspended in 12 mL lysis buffer (50mM Hepes pH 8.0, 400mM NaCl, 10% glycerol, 3mM imidazole, 0.5mM PMSF, 4mM β-mercaptoethanol, 1 tablet/10mL c0mplete Mini EDTA-free Protease Inhibitor Cocktail (Roche)) and lysed by sonication. The soluble fraction obtained after centrifugation (20.000xg for 20 minutes at 4°C) was incubated for 90 minutes at 4°C with 250 µL of Ni-NTA-agarose (Qiagen) that was pre-washed with lysis buffer. This allowed binding of the BTB domain to the beads via the His-tag. After incubation the beads were washed using 50 mL wash buffer (25mM Hepes pH 8.0, 300mM NaCl, 10% glycerol, 10mM imidazole, 0.2mM PMSF, 5mM β-mercaptoethanol, 10µg/mL apropotin/leupeptin). The BTB domain was eluted in elution buffer (25mM Hepes pH 8.0, 200mM NaCl, 10% glycerol, 300mM imidazole, 5mM β-mercaptoethanol, 10µg/mL apropotin/leupeptin). The elution was loaded in a Slide-A-Lyzer® Dialysis Cassette 3,500 MWCO 0.5 – 3 ml Capacity (Thermo Scientific) and dialyzed for one hour and again overnight in 500 mL dialysis buffer (25mM Hepes pH 8.0, 200mM NaCl, 10% glycerol, 5mM β-mercaptoethanol) for the removal of imidazole. The protein was aliquoted and stored at -80°C.

Elisa

To detect the disappearance of CPDs in Xenopus laevis egg extract, 6 ng/ul non-treated or UV-C treated plasmid DNA was incubated in a 6,25 µl reaction containing 2,5 µl HSS, supplemented with 5mM $MgCl_2$, 0.5mM DTT, 4mM ATP, 40mM Phosphocreatine and 0.5µg creatine phosphokinase. Reactions were incubated at room temperature and stopped by addition of ten volumes of Stop Solution II (0.5% SDS, 10 mM EDTA, 50 mM Tris pH 7.5) at 0 or 2 hours. Samples were incubated with Proteinase K (0.5 µg/µl) for 30 min at 37°C. DNA was extracted using Phenol/Chloroform, ethanol precipitated in the presence of glycogen (30 mg/ml), and resuspended in 5-10 µl of 10 mM Tris pH 7.5. CPDs were detected using OxiSelect UV-Induced DNA Damage ELISA Kit (Bioconnect) according to manufacturer's instructions.

Size exclusion chromatography

In order to assess the binding between XPF and SLX4-BTB, purified proteins were loaded onto a Superdex 200 increase 10/300 GL gel-filtration column (GE Healthcare). Samples were taken at different elution volumes and analyzed by western blot using respective antibodies.

Antibodies and immunodepletions

Antibodies against *xl*PCNA and *xl*XPA were previously described (Kochaniak *et al.*, 2009; Bomgarden RD Soni DV, Yee MC, Ford JM, Cimprich KA. *et al.*, 2006). PCNA was removed from the HSS extract using 6 rounds of depletion for 30 min or 1h at 4 °C using Protein A Sepharose Fast Flow (PAS) (GE Healthcare) bound to *xl*PCNA antibody (1 volume of PAS was bound to 3 volumes of anti-serum or pre-immune serum, and added to 5 volumes of HSS). For the XPA depletion one volume of PAS was bound to one volume of anti-serum or pre-immune serum and added to 4 volumes of HSS for 30 min at 4°C. This was repeated 4 times.

Appendix references

Bomgarden RD Soni DV, Yee MC, Ford JM, Cimprich KA. LPJ, Bomgarden RD, Lupardus PJ, Soni D V, Yee M-C, Ford JM & Cimprich K a (2006) Opposing effects of the UV lesion repair protein XPA and UV bypass polymerase eta on ATR checkpoint signaling. *EMBO J.* **25:** 2605–2614

Kochaniak AB, Habuchi S, Loparo JJ, Chang DJ, Cimprich KA, Walter JC & van Oijen AM (2009) Proliferating cell nuclear antigen uses two distinct modes to move along DNA. *J. Biol. Chem.* **284:** 17700–10
