# Supplementary material for: Recruitment and positioning determine the specific role of the XPF‐ERCC1 endonuclease in interstrand crosslink repair
Source: EMBO J. 2017 Mar 14;36(14):2034–46. doi: 10.15252/embj.201695223 (PMC5510004; doi:10.15252/embj.201695223)
Supplement: Supplementary file 2 — Expanded View Figures PDF [file EMBJ-36-2034-s002.pdf]

Expanded View Figures

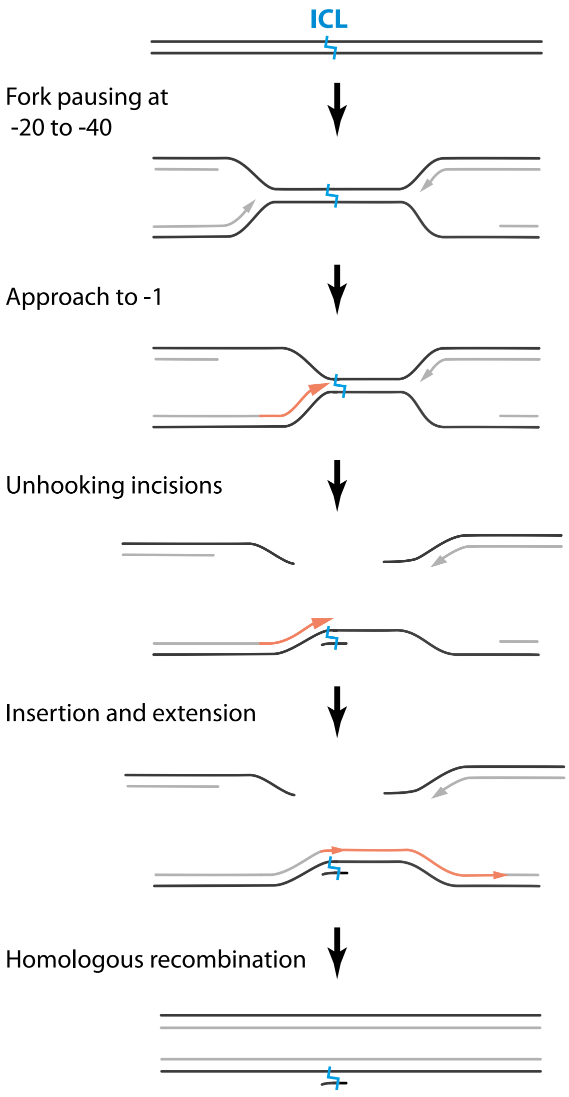

Figure EV1. Schematic representation of replication-dependent ICL repair in *Xenopus* egg extract.
Arrow heads represent 3' ends of leading strands.

 © 2017 The Authors

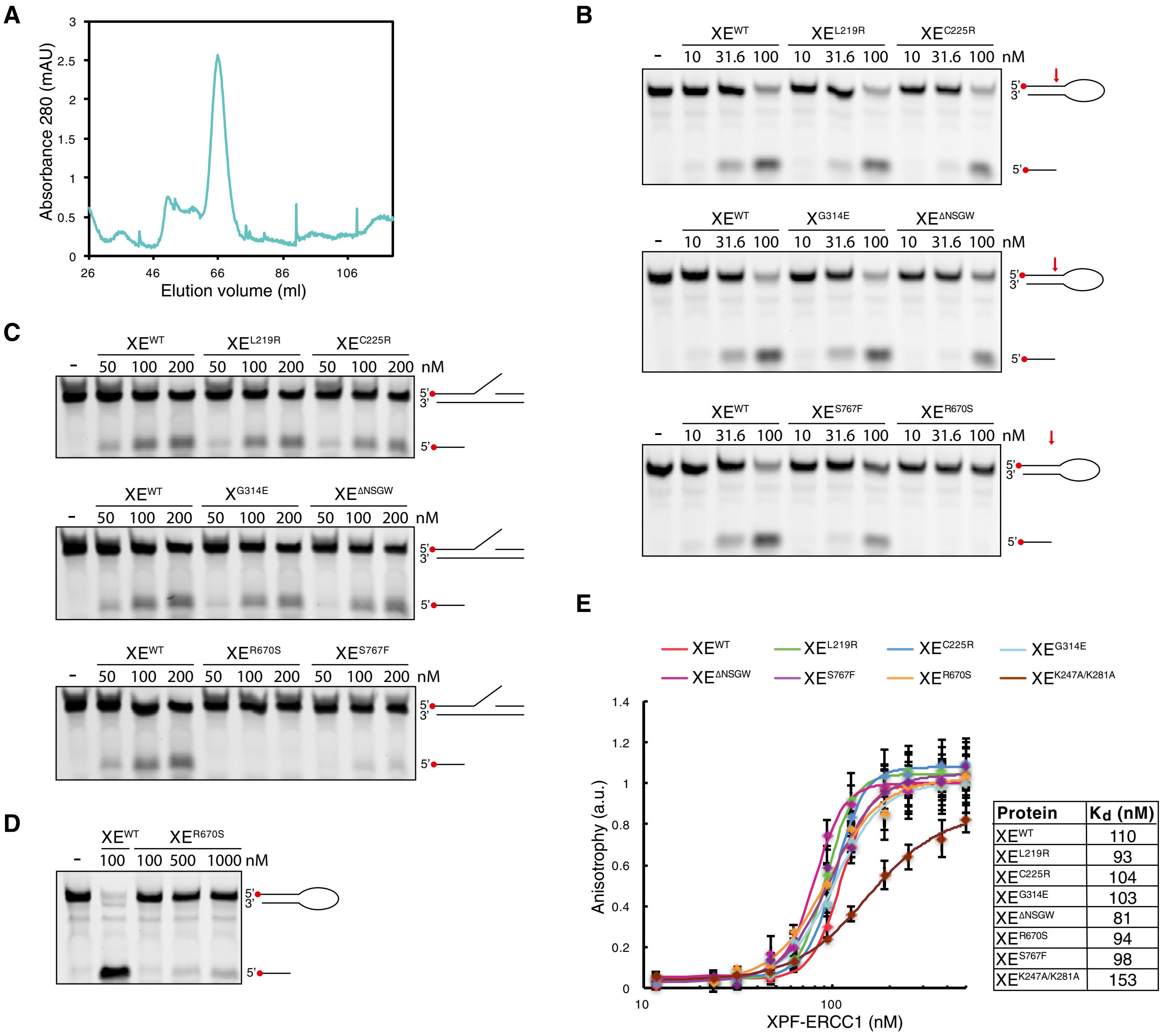

Figure EV2. Characterization of mutant XPF-ERCC1 complexes.

A Superdex 200 gel filtration column elution profile of XPF^{L219R}-ERCC1. The heterodimeric fraction depicted in Fig 1A was collected, concentrated, and rerun on the same column. The protein did not aggregate and eluted as a heterodimer at ~65 ml.

B Replicate of Fig 1D. Wild-type and indicated mutant XPF-ERCC1 complexes were incubated with a 5′-FAM-labeled stem-loop DNA substrate (10 nM) at room temperature for 30 min. Reaction products were separated on a 12% urea–PAGE gel and visualized using a fluorescence imaging system. Red arrow indicates position of incision by XPF-ERCC1.

C As in (B) but using a 5′-FAM-labeled 3′ flap DNA substrate.

D As in (B) but using higher concentrations of the XE^{R670S} mutant.

E Replicate of Fig 1E. Wild-type and mutant XPF-ERCC1 complexes at various concentrations were incubated with a 5′-FAM-labeled 3′ flap DNA substrate (10 nM) and fluorescent anisotropy was measured. Graphs were fitted to calculate dissociation constants (K_ds) as described in the Materials and Methods section. The error bars represent s.d. from three measurements.

Source data are available online for this figure.

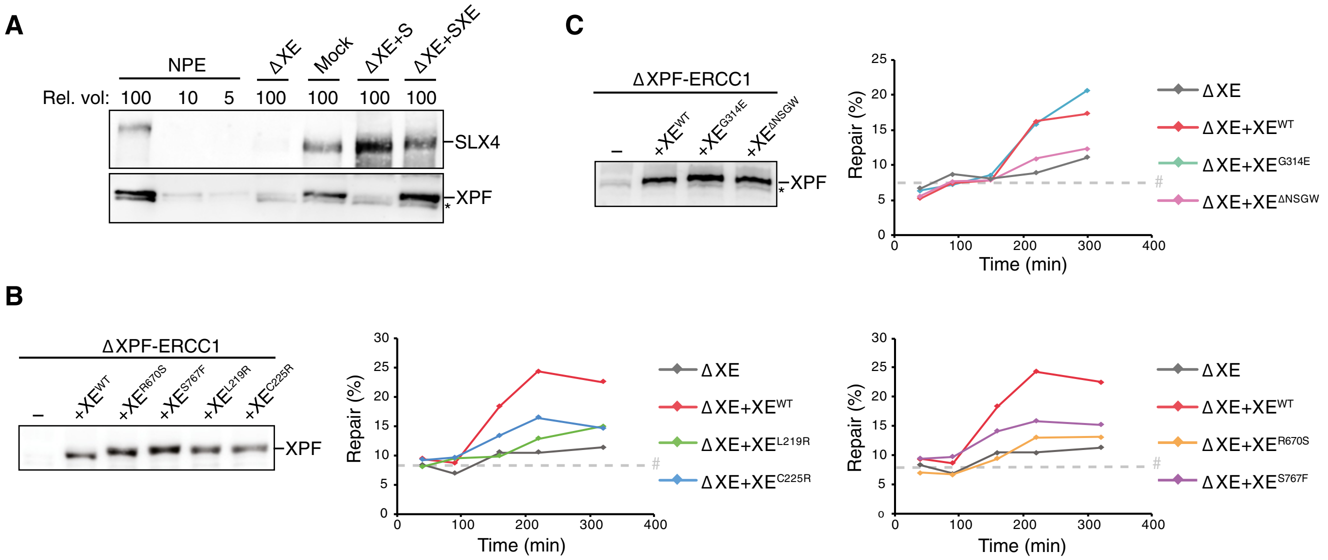

Figure EV3. Effect of mutations in XPF-ERCC1 on ICL repair in *Xenopus* egg extract.

A Mock-depleted, XPF-ERCC1-depleted (ΔXE), and XPF-ERCC1-depleted NPE complemented with SLX4 (ΔXE+S) or XPF-ERCC1 and SLX4 (ΔXE+SXE) were analyzed by Western blot using α-XPF or α-SLX4 antibodies. A dilution series of undepleted NPE was loaded on the same blot to determine the degree of depletion. A relative volume of 100 corresponds to 0.2 μl of NPE.

B Replicates of Fig 2B. XPF-ERCC1-depleted (ΔXE) and XPF-ERCC1-depleted extracts complemented with wild-type (XE^{WT}) or indicated mutant XPF-ERCC1 (XE^{MUT}) were analyzed by Western blot using α-XPF antibodies (left panel). These extracts were used to replicate pICL. Replication intermediates were isolated and digested with HincII, or HincII and SapI, and separated on an agarose gel. Repair efficiency was calculated and plotted (right panels).

C As in (B) but analyzing different mutant complexes. Note: repair levels can differ per batch of individually prepared extract or per depletion experiment and can only be compared within an experiment.

Data information: (B, C) #, SapI fragments from contaminating uncrosslinked plasmid present in varying degrees in pICL preparations. (A, C) *, background band.

Source data are available online for this figure.

 © 2017 The Authors

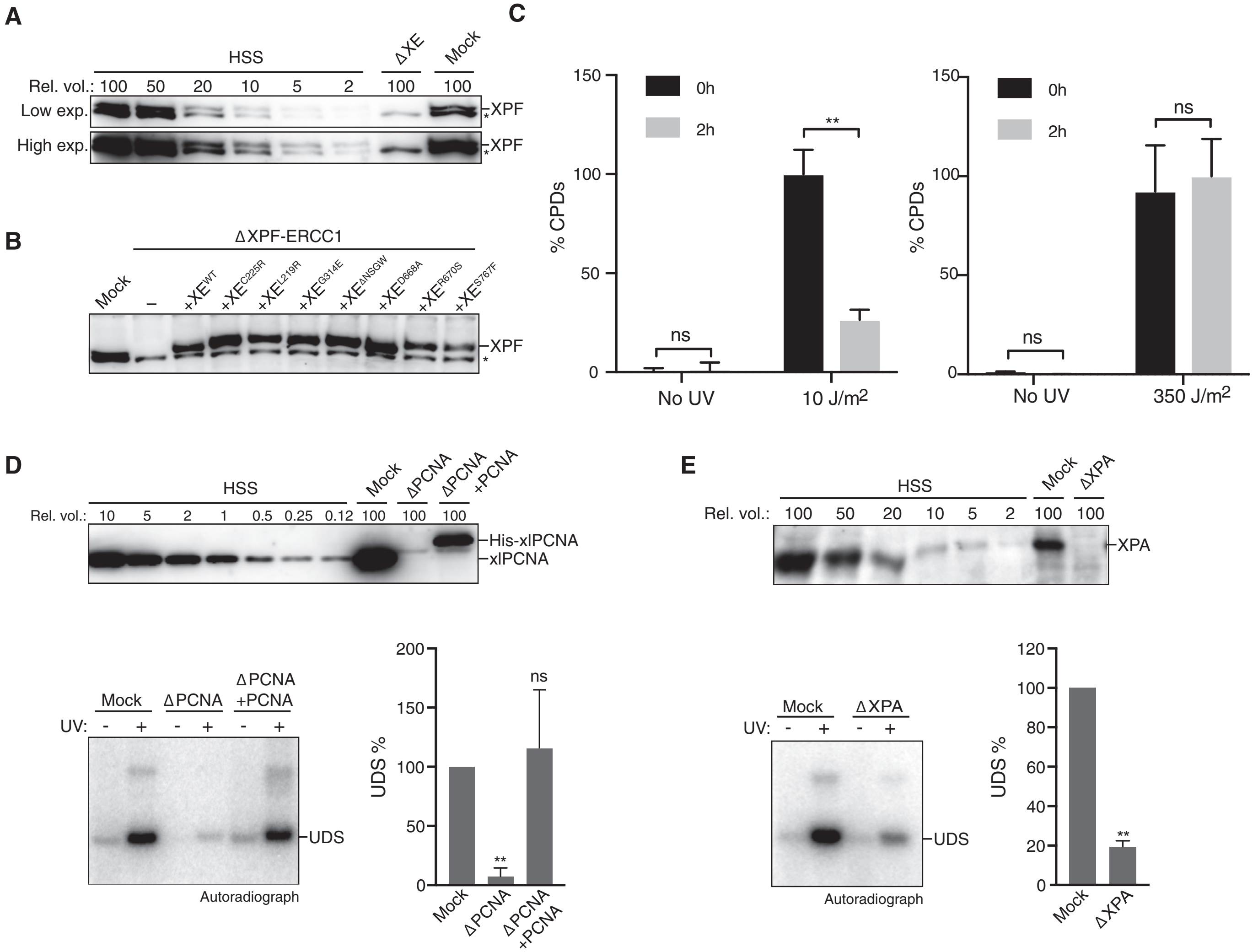

Figure EV4. XPF-ERCC1 mutant complexes are active in NER.

A Mock-depleted and XPF-ERCC1-depleted (ΔXE) high-speed supernatant (HSS) egg extracts used in Fig 3B were analyzed by Western blot using α-XPF antibodies. A dilution series of undepleted NPE was loaded on the same blot to determine the degree of depletion. A relative volume of 100 corresponds to 0.2 μl of NPE. *, background band.

B Mock-depleted, XPF-ERCC1-depleted (ΔXE), and XPF-ERCC1-depleted HSS complemented with wild-type (XE^{WT}) or mutant XPF-ERCC1 (XE^{MUT}) were analyzed by Western blot using α-XPF antibodies. *, background band.

C Untreated or UV-treated (10 J/m^2, left panel, 350 J/m^2 right panel) plasmid DNA was incubated in HSS for 2 h. Samples were taken at time 0 and 2 h, and DNA was extracted and analyzed by an enzyme-linked immunosorbent assay (ELISA) for the presence of CPDs. The highest value within one experiment was set to 100%. Error bars represent s.e.m. of three independent experiments. **P = 0.0061, paired t-test. ns, not significant.

D Mock-depleted, PCNA-depleted (ΔPCNA), and PCNA-depleted HSS complemented with recombinant His-*xl*PCNA (ΔPCNA + PCNA) were analyzed by Western blot using α-PCNA antibodies (Kochaniak *et al*, 2009). A dilution series of undepleted NPE was loaded on the same blot to determine the degree of depletion. A relative volume of 100 corresponds to 0.2 μl of NPE (top panel). These extracts were incubated with untreated or UV-treated (350 J/m^2) plasmids for 0 or 2 h at room temperature in the presence of ^{32}P-α-dCTP. Reaction products were isolated, linearized with HincII, and separated on a 0.8% agarose gel. The DNA was visualized by autoradiography to show incorporation of ^{32}P-α-dCTP during UDS (bottom left panel). The signal was quantified, the background signal from non-damaged plasmid was subtracted, and the signal for the mock depletion condition was set to 100% to normalize the data. Error bars represent s.e.m. of three independent experiments. **P = 0.0059, paired t-test compared to the mock condition. ns, not significant.

E As in (D) but using mock-depleted or XPA-depleted HSS. **P = 0.0014.

Source data are available online for this figure.

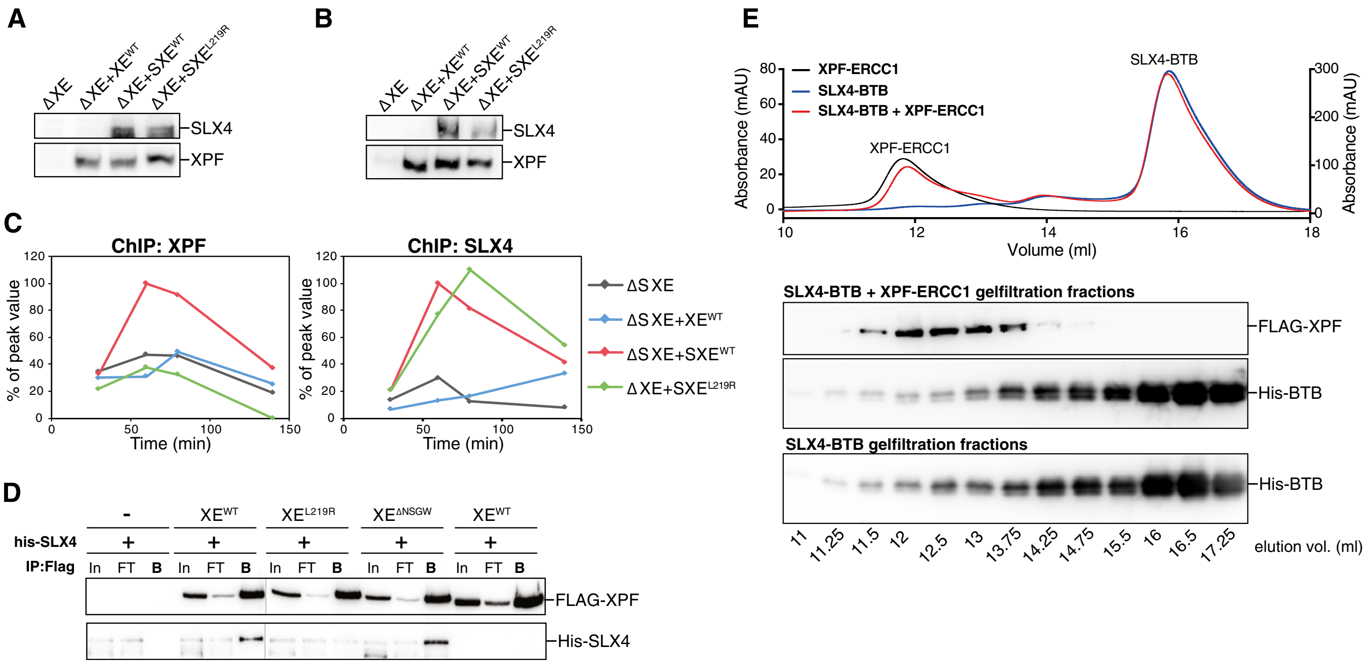

Figure EV5. XPF leucine 219 is part of the major interaction site between XPF and SLX4.

A XPF-ERCC1-depleted (ΔXE) and XPF-ERCC1-depleted NPE supplemented with XPF-ERCC1 (+XE^{WT}), XPF-ERCC1 and SLX4 (+SXE^{WT}), or XPF^{L219R}-ERCC1 and SLX4 (+SXE^{L219R}) were analyzed by Western blot using α-XPF and α-SLX4 antibodies. Extracts were used for Fig 6A.

B As in (A).

C Replicate of Fig 6A. The extracts from (B), with similarly treated HSS, were used to replicate pICL. Samples were taken at the indicated times and analyzed by XPF (left) and SLX4 (right) ChIP using pICL and pQuant primers. The qPCR data were plotted as the percentage of peak value with the highest value set to 100%.

D Replicate of Fig 6C. Wild-type and mutant FLAG-XPF-ERCC1 were co-expressed with His-SLX4 in Sf9 insect cells. Cells were lysed and XPF was immunoprecipitated via the FLAG-tag. Samples were analyzed by Western blot using α-FLAG and α-His antibodies. In, input; FT, flow-through fraction; B, fraction bound to beads.

E Size exclusion chromatography of recombinant XPF-ERCC1 and BTB domain of SLX4. Superdex 200 gel filtration column elution profile of FLAG-XPF-ERCC1, His-tagged BTB domain, and both proteins combined (top panel). The XPF-ERCC1 heterodimer eluted at ~12 ml, while His-BTB eluted around ~16 ml. Collected fractions during elution were analyzed by Western blot using α-XPF and α-His antibodies (bottom panel). The BTB domain protein does not shift to a higher elution volume when incubated with XPF-ERCC1 indicating the affinity is not high enough to show binding between the two proteins.

Source data are available online for this figure.

 © 2017 The Authors
