# Supplementary material for: Recruitment and positioning determine the specific role of the XPF‐ERCC1 endonuclease in interstrand crosslink repair
Source: EMBO J. 2017 Mar 14;36(14):2034–46. doi: 10.15252/embj.201695223 (PMC5510004; doi:10.15252/embj.201695223)
Supplement: Supplementary file 3 — Source Data for Expanded View and Appendix [file EMBJ-36-2034-s003.zip › EMBOJ_95223_Source_Data/FigEV4.pdf]

Source data Fig EV4

A

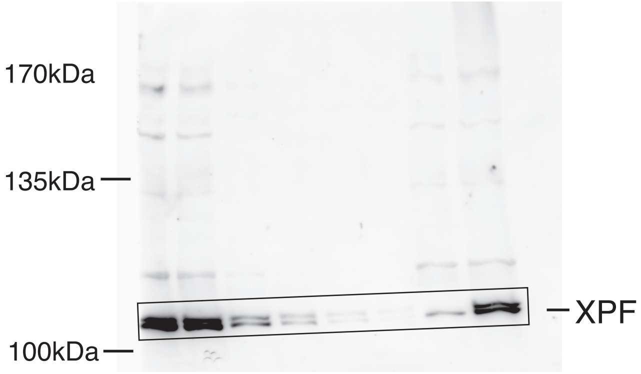

B

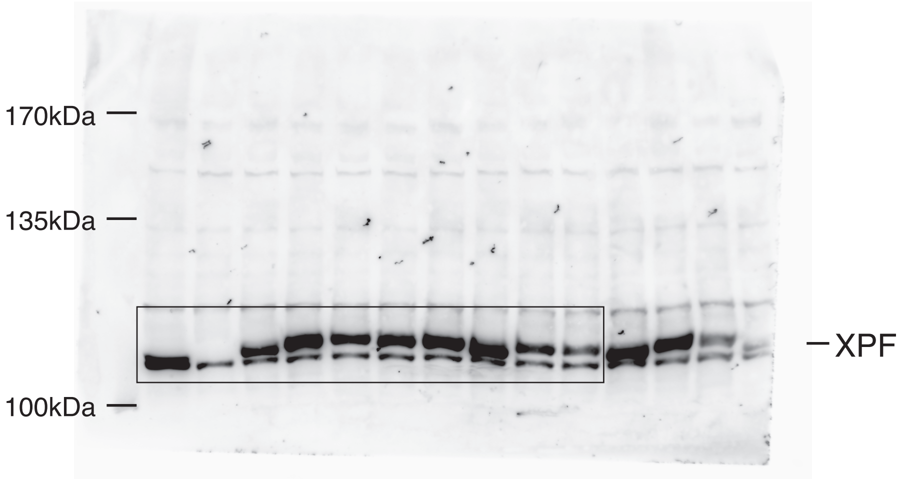

D

Top panel

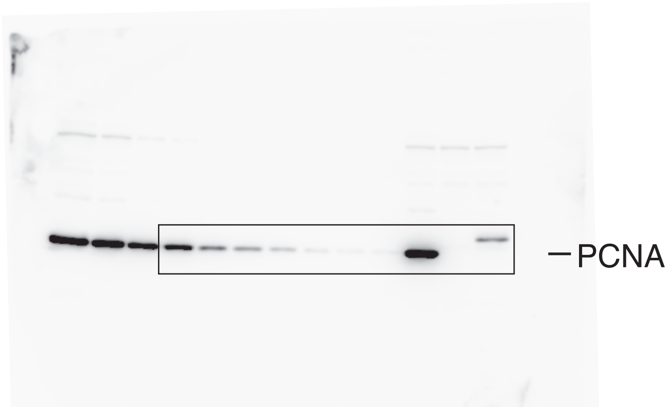

Light

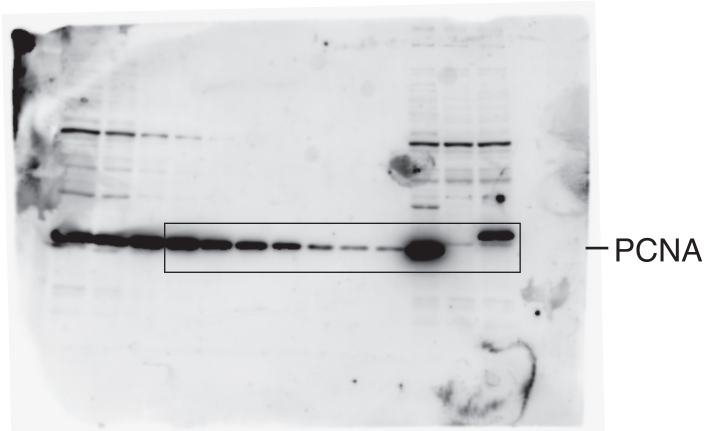

Dark

Including colorimetric marker overlay from AI 600 imager (GE)

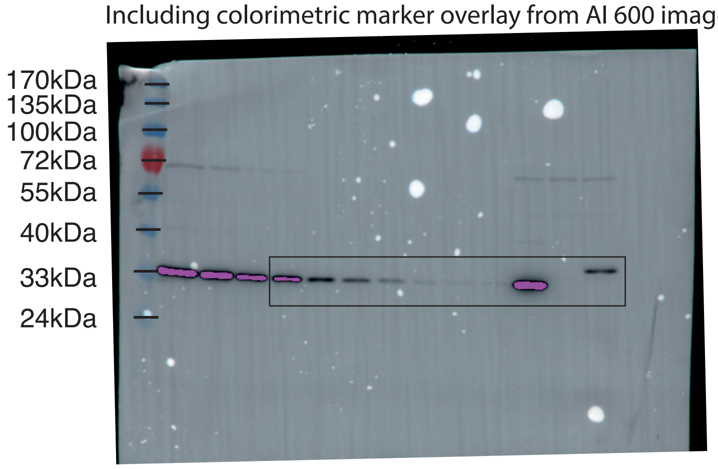

Bottom panel

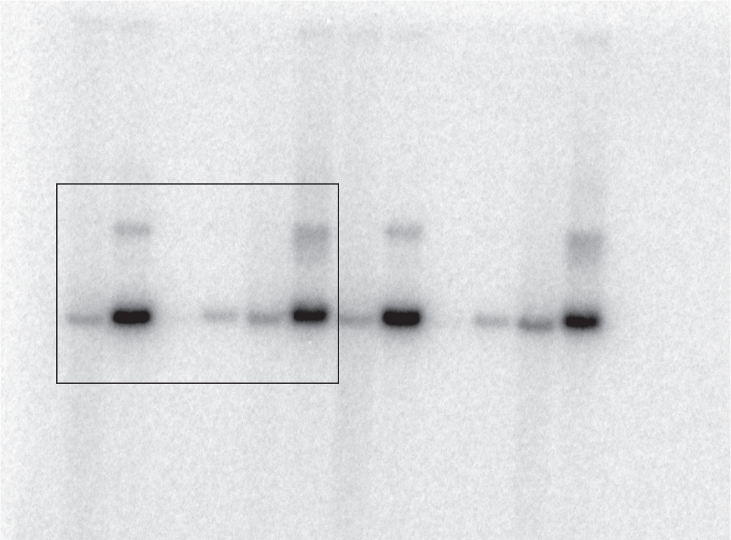

Autoradiograph

E

Top panel

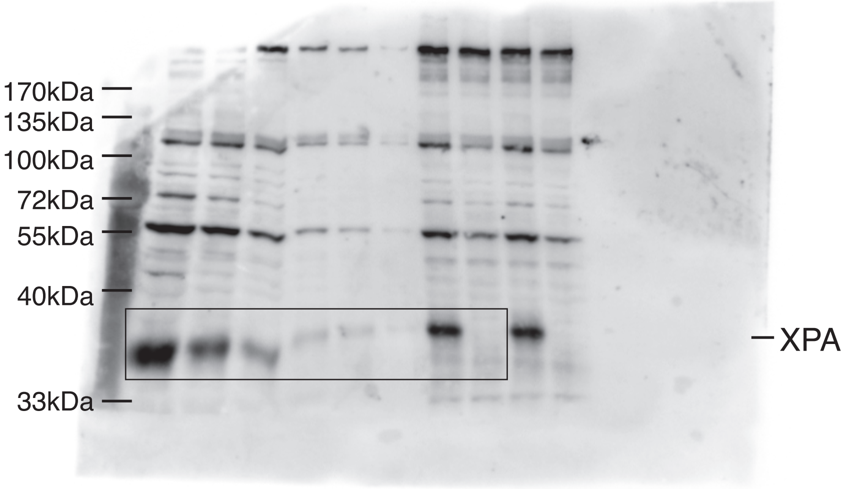

Bottom panel

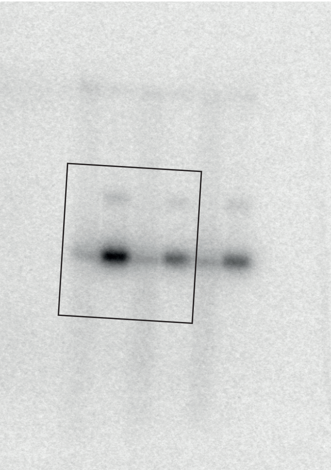

Autoradiograph
