# Supplementary figures and images for: Recruitment and positioning determine the specific role of the XPF‐ERCC1 endonuclease in interstrand crosslink repair
Source: EMBO J. 2017 Mar 14;36(14):2034–46. doi: 10.15252/embj.201695223 (PMC5510004; doi:10.15252/embj.201695223)

Source data Appendix Fig S1A

left

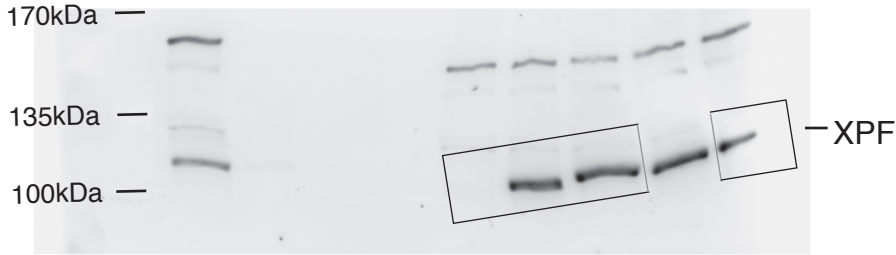

middle

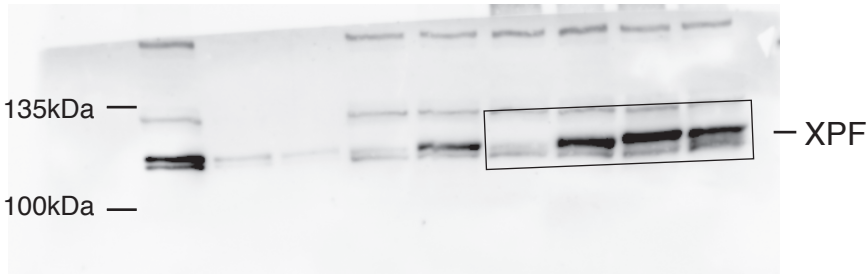

right

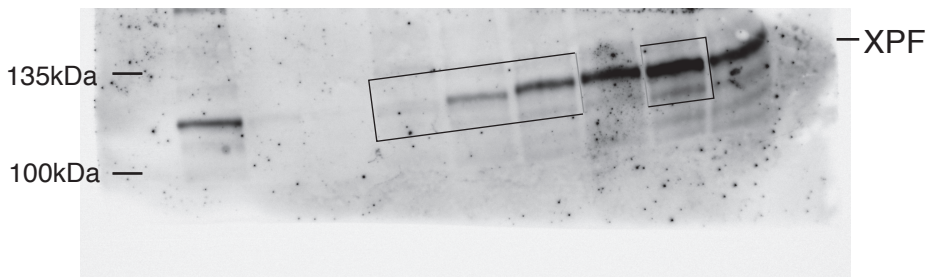

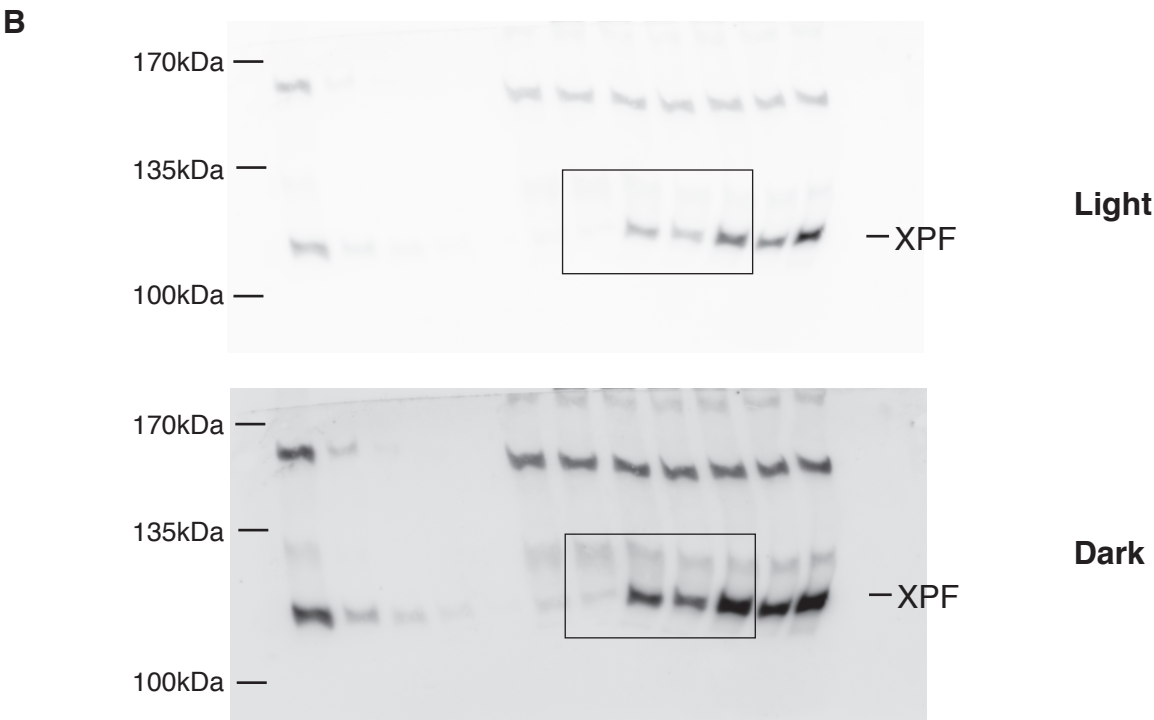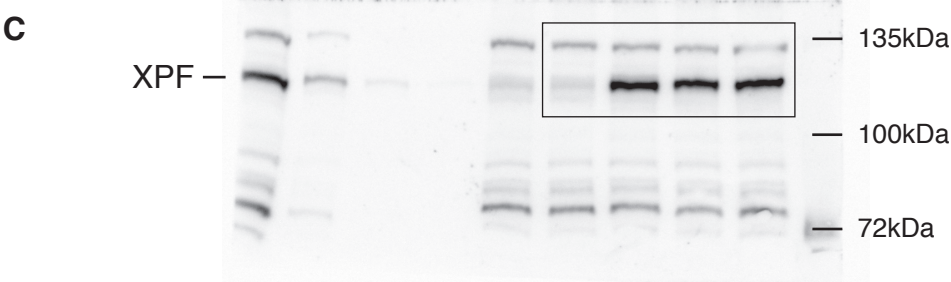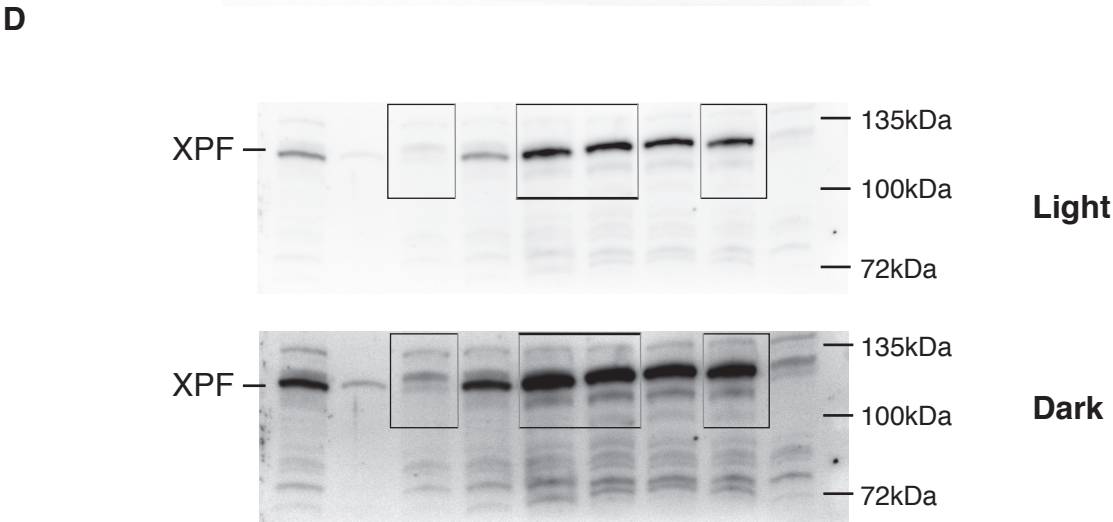

Supplement: Supplementary file 3 — Source Data for Expanded View and Appendix [file EMBJ-36-2034-s003.zip › EMBOJ_95223_Source_Data/AppendixFigS1.pdf]

Appendix Figure S2

**A**

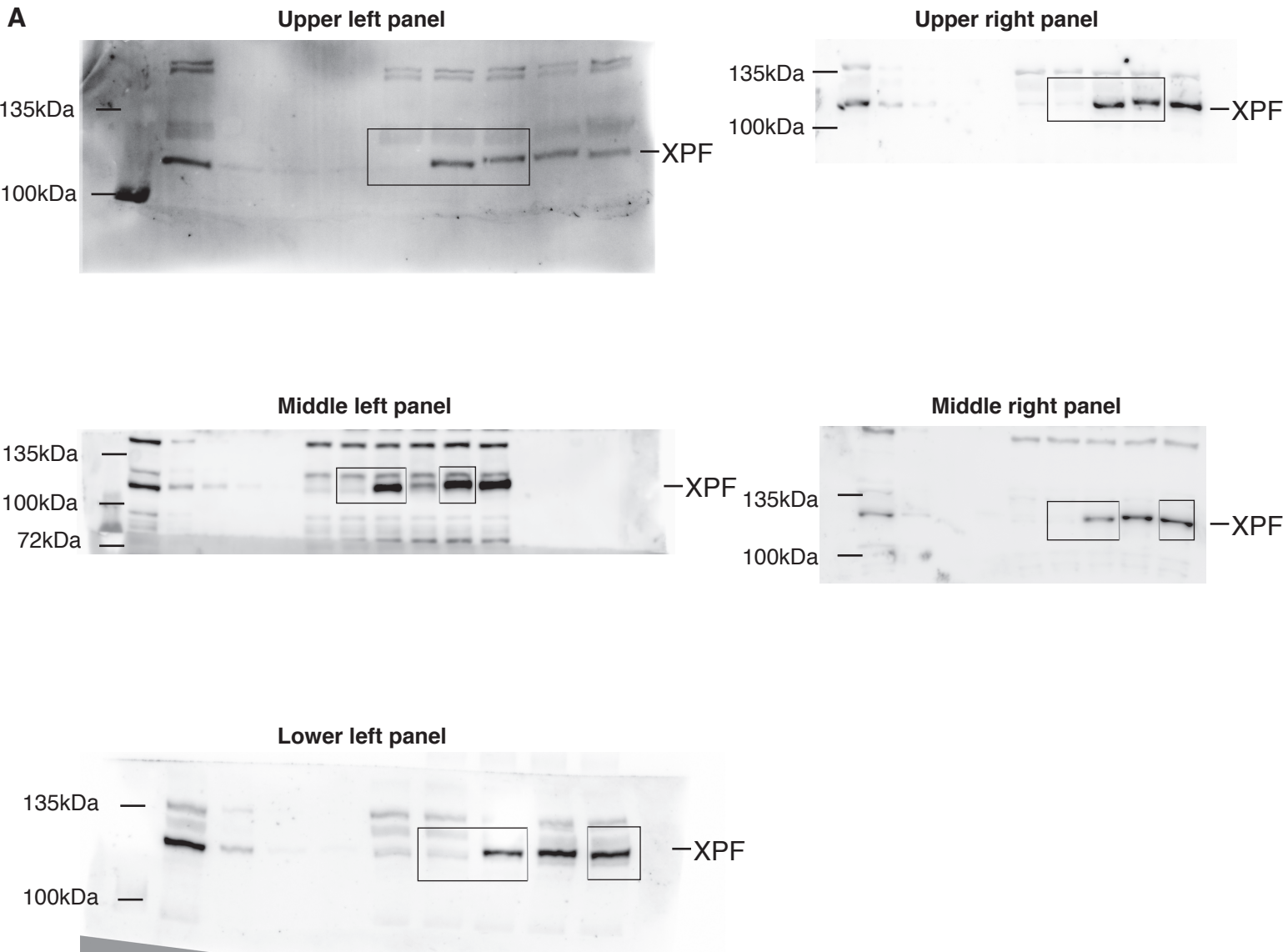

**B**

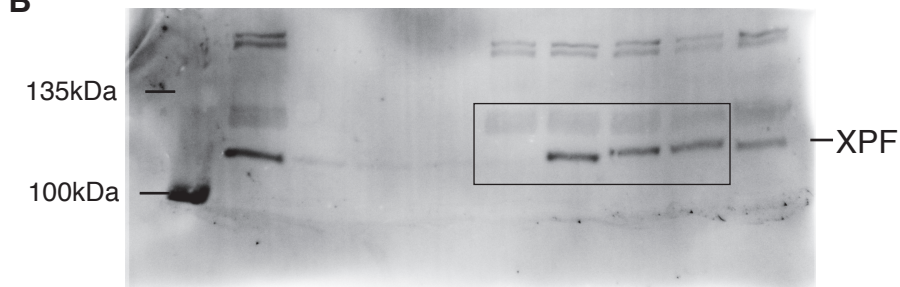

**C**

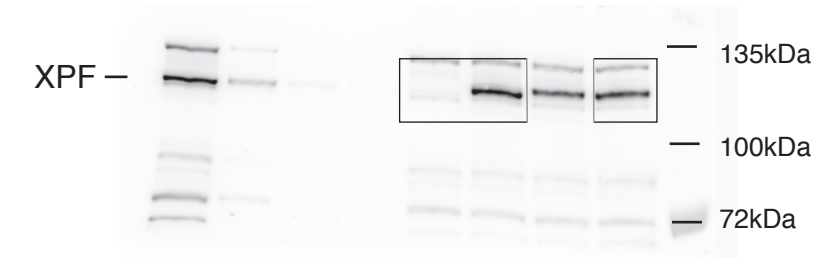

**D**

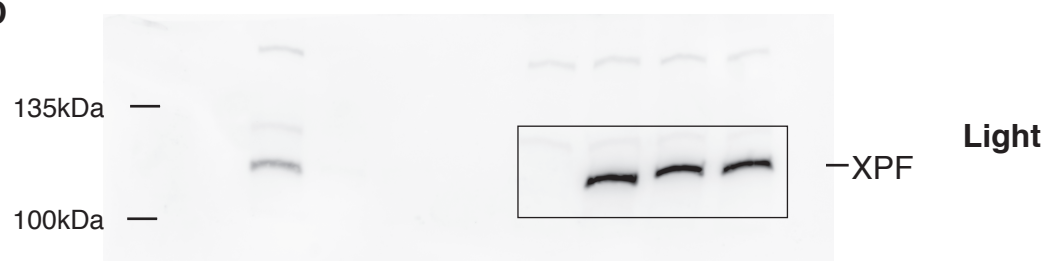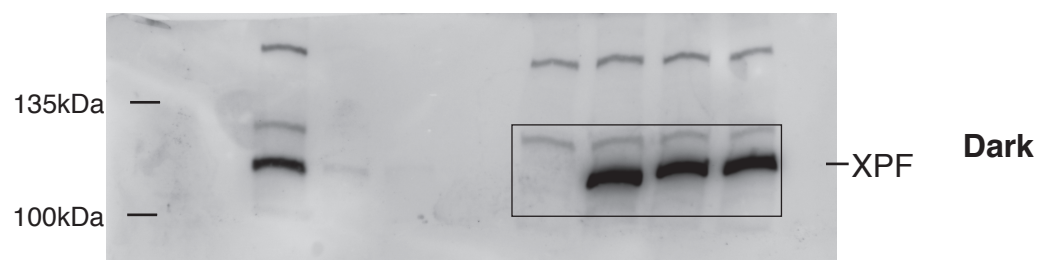

Supplement: Supplementary file 3 — Source Data for Expanded View and Appendix [file EMBJ-36-2034-s003.zip › EMBOJ_95223_Source_Data/AppendixFigS2.pdf]

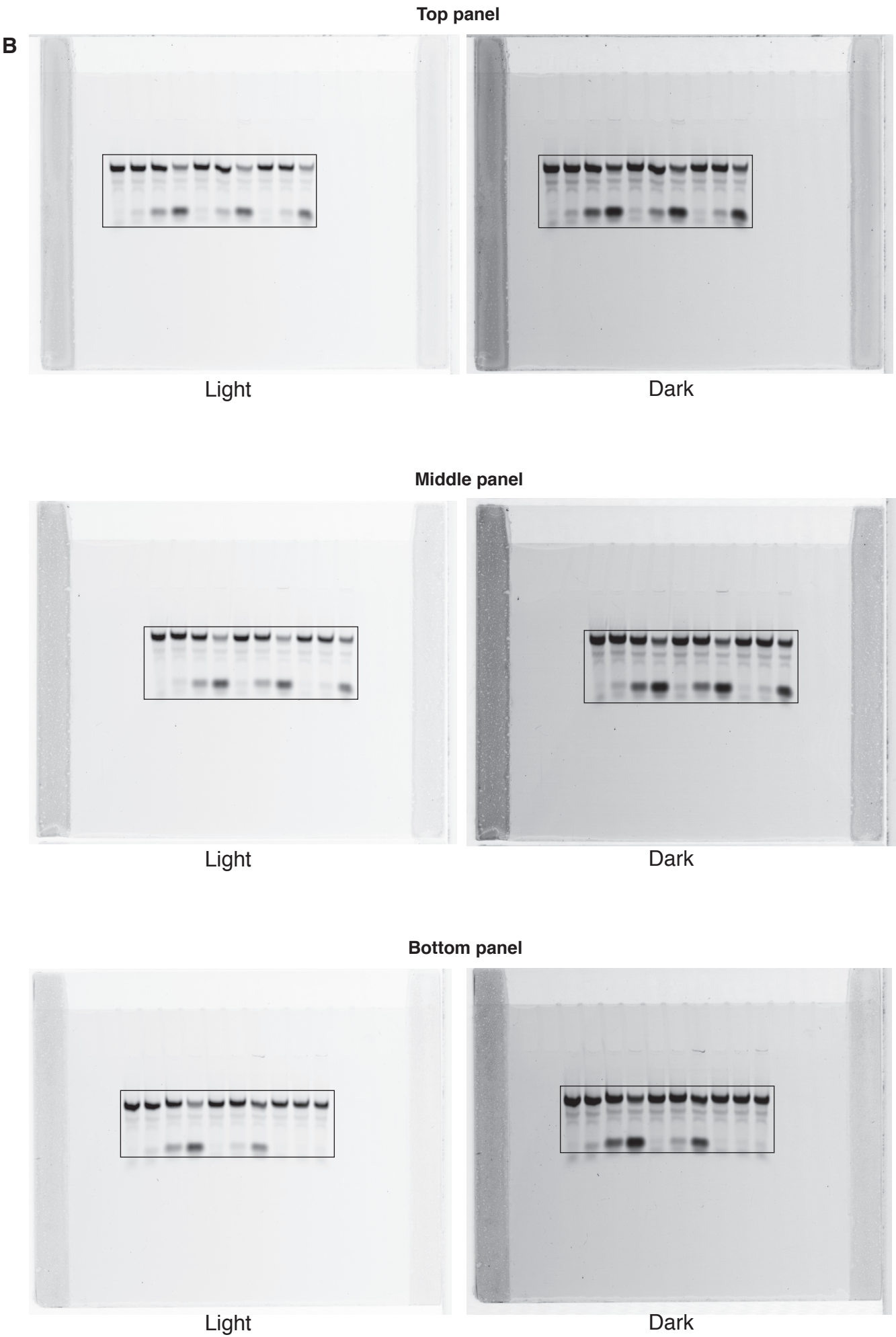

**C**

**Top panel**

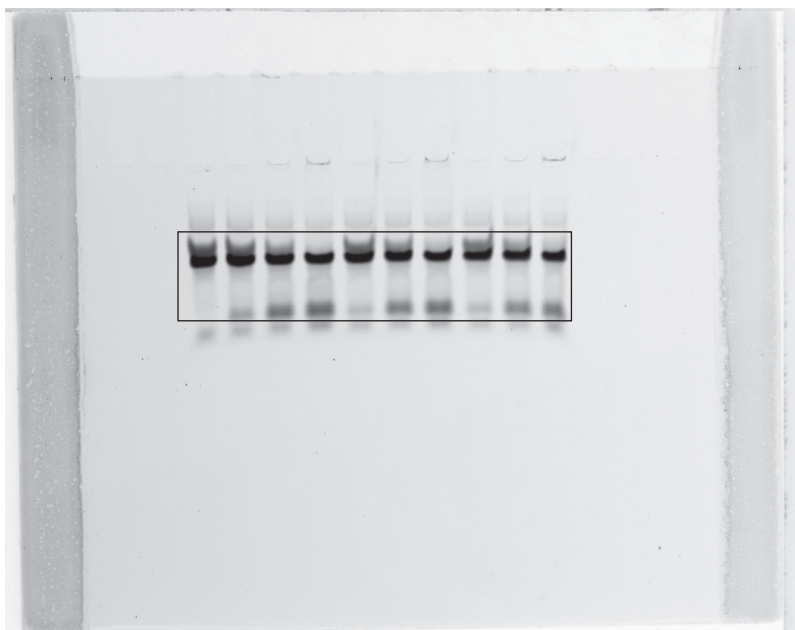

**Middle panel**

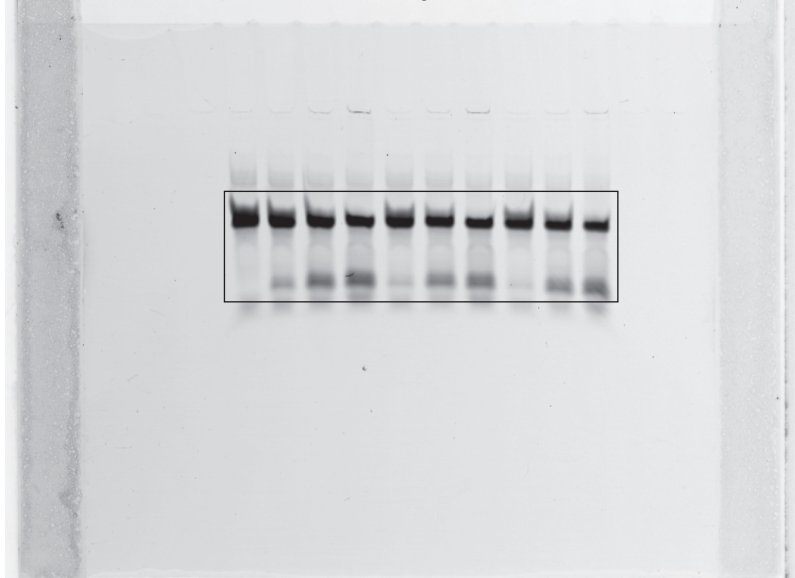

**Bottom panel**

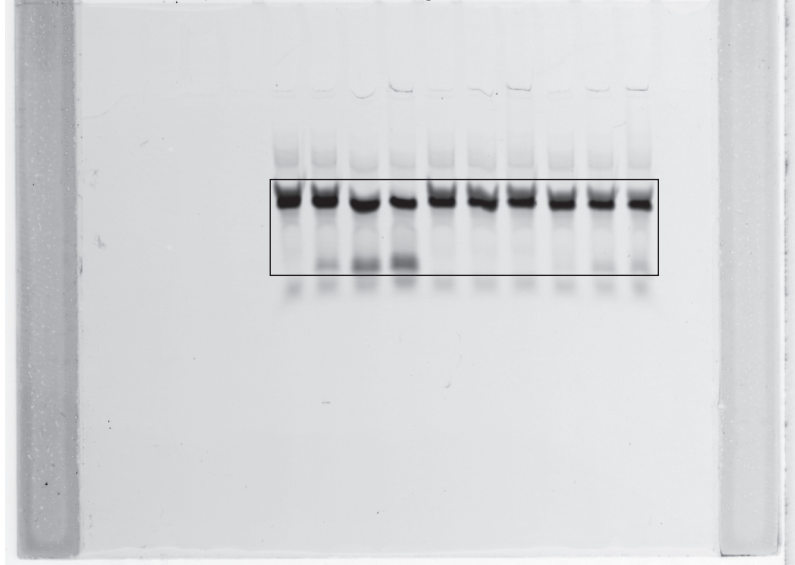

**D**

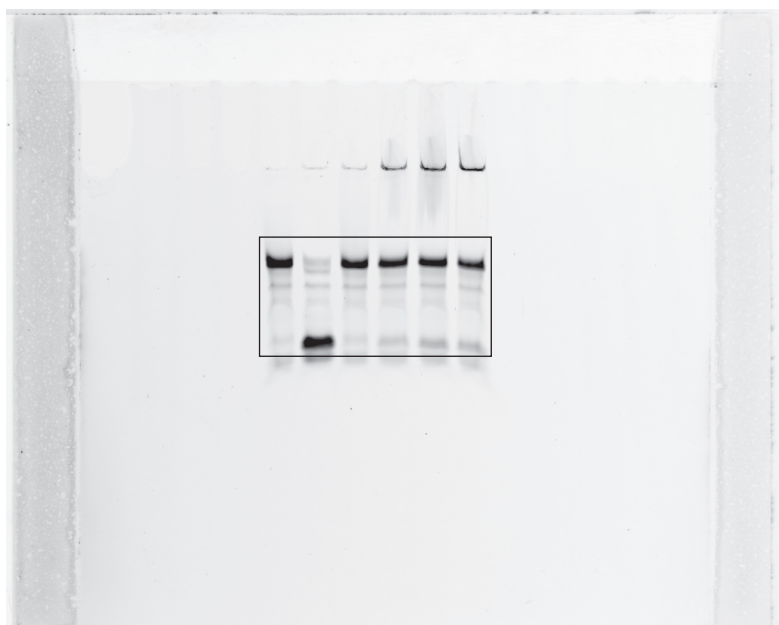

Supplement: Supplementary file 3 — Source Data for Expanded View and Appendix [file EMBJ-36-2034-s003.zip › EMBOJ_95223_Source_Data/FigEV2.pdf]

Source data Fig EV3

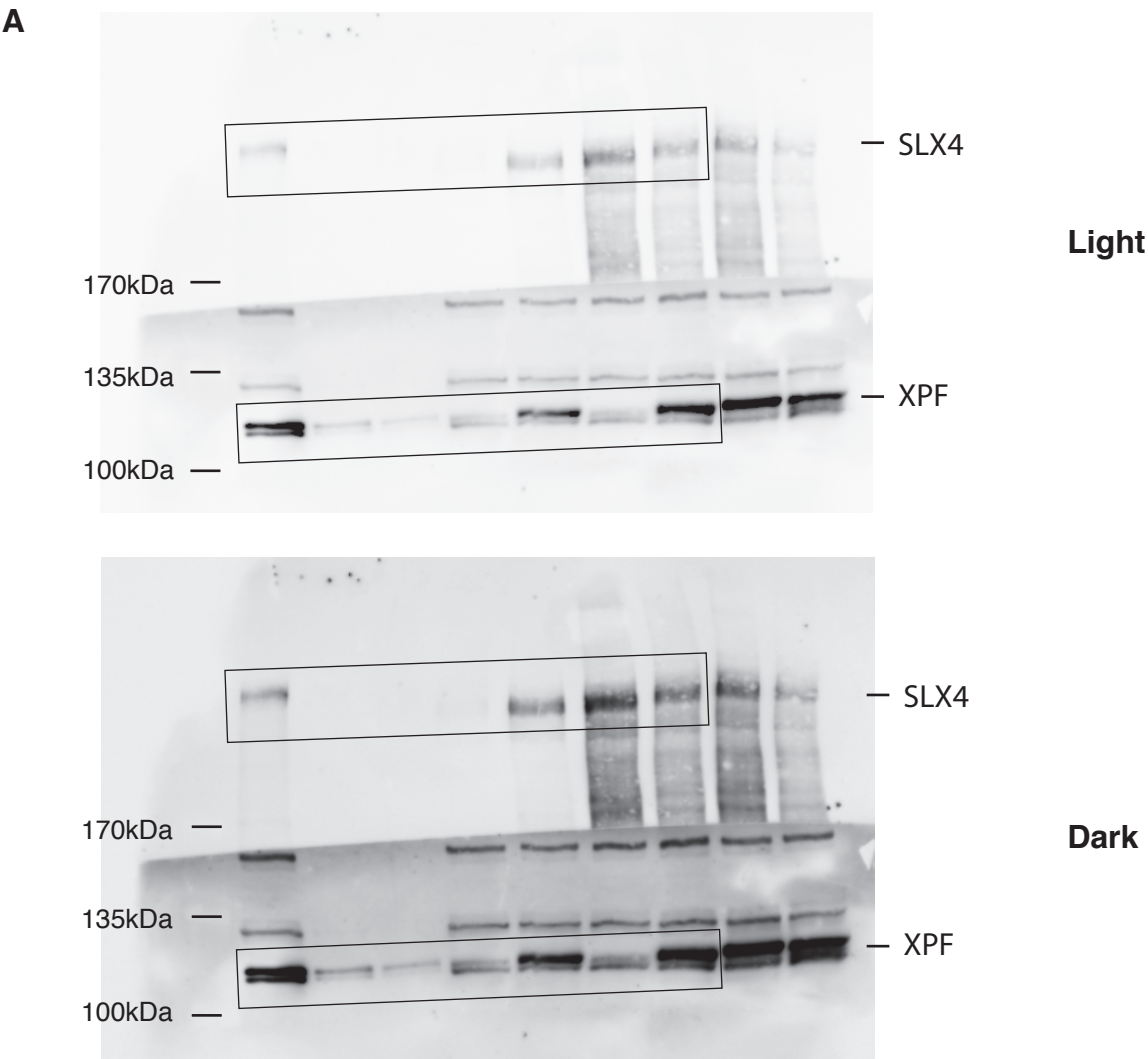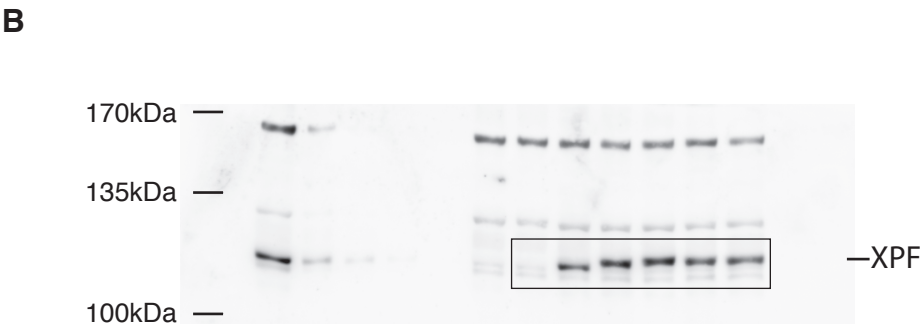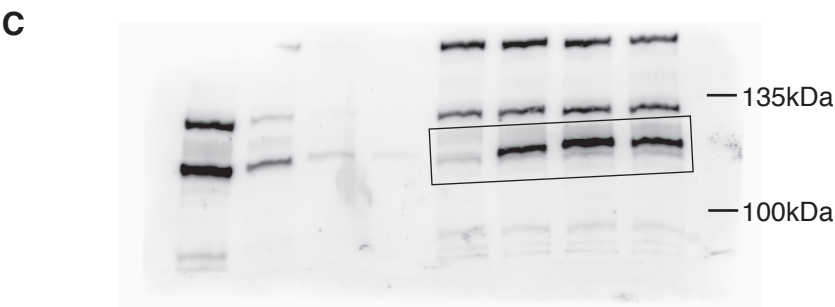

Supplement: Supplementary file 3 — Source Data for Expanded View and Appendix [file EMBJ-36-2034-s003.zip › EMBOJ_95223_Source_Data/FigEV3.pdf]

Source data Fig EV5

**A**

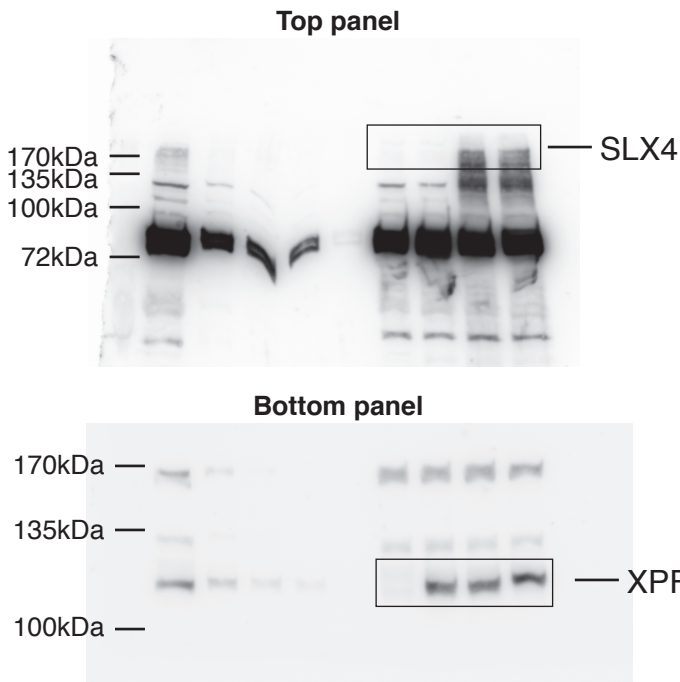

**B**

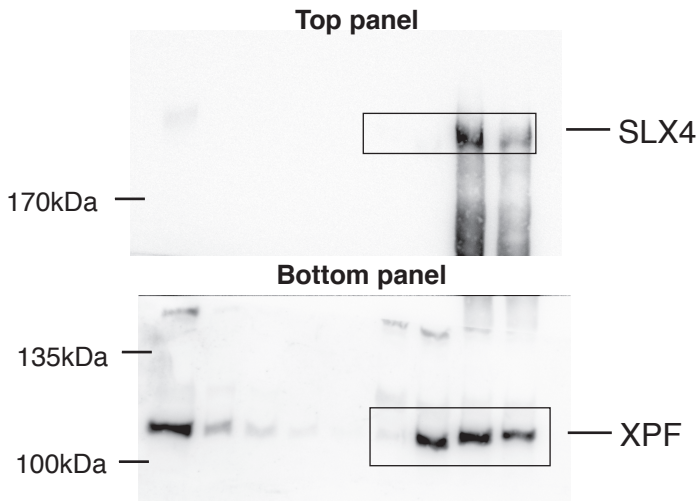

**D**

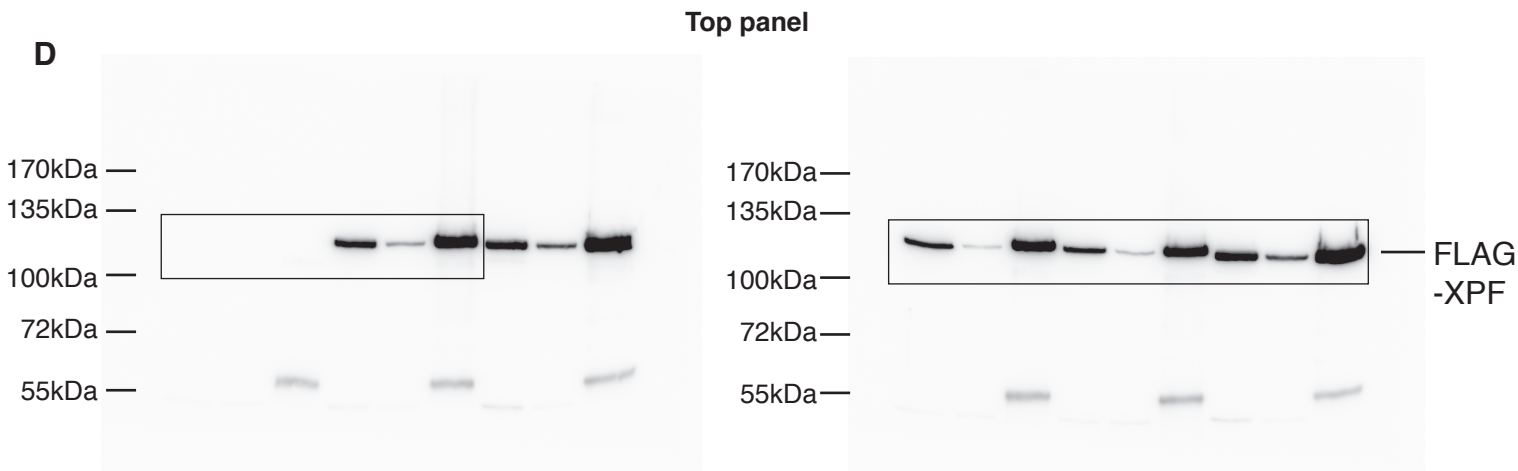

**Bottom panel**

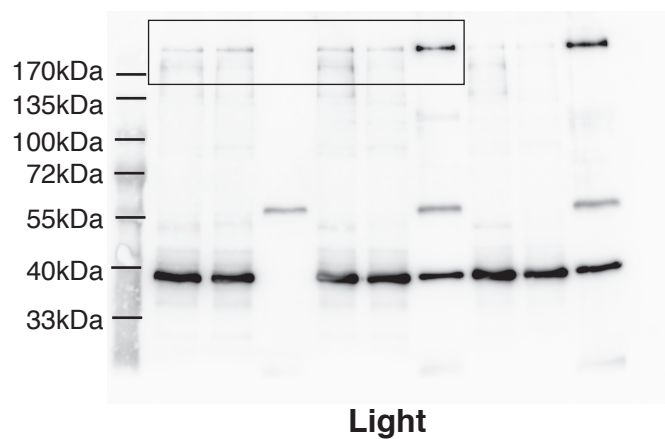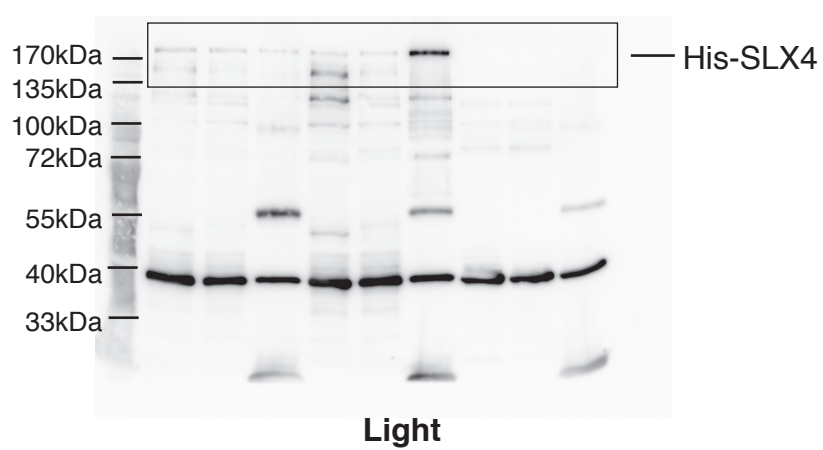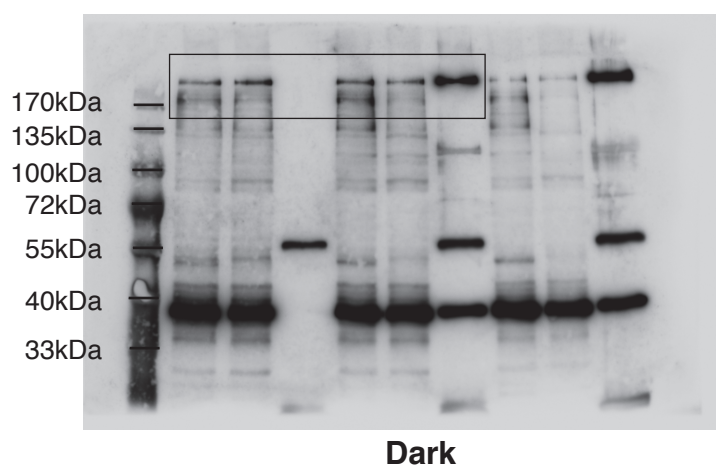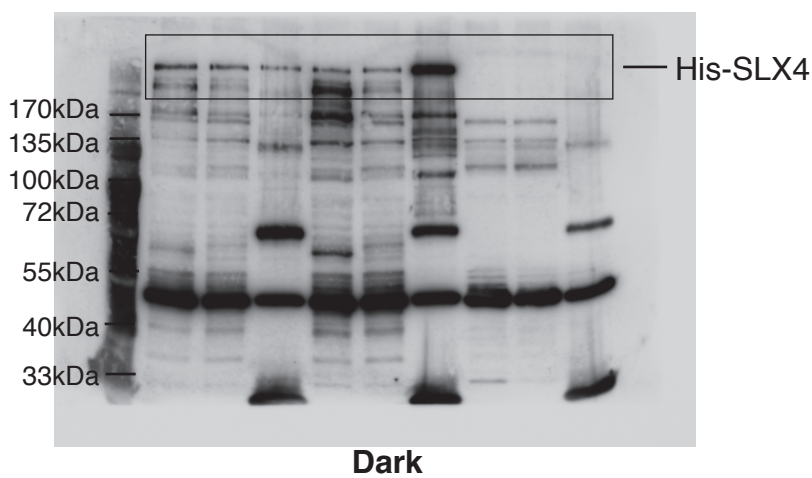

Supplement: Supplementary file 3 — Source Data for Expanded View and Appendix [file EMBJ-36-2034-s003.zip › EMBOJ_95223_Source_Data/FigEV5.pdf]

# Source data Fig 1

**B**

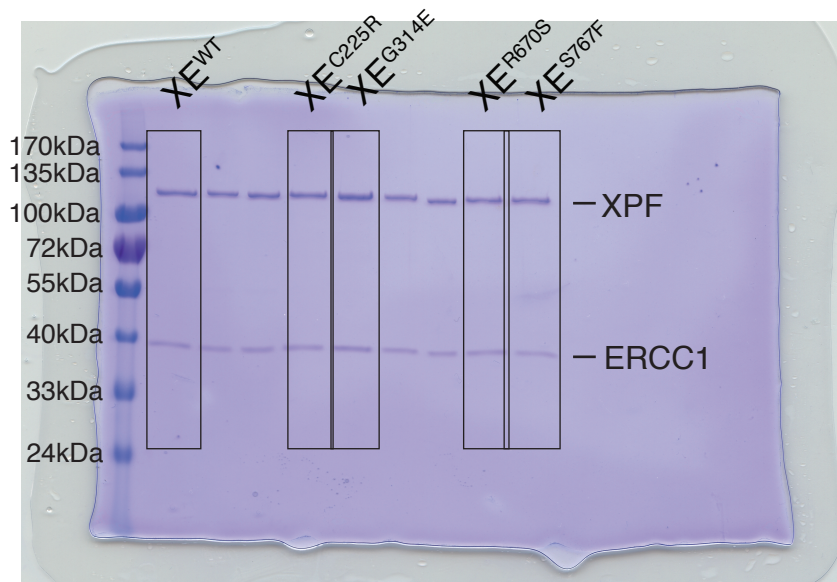

**C**

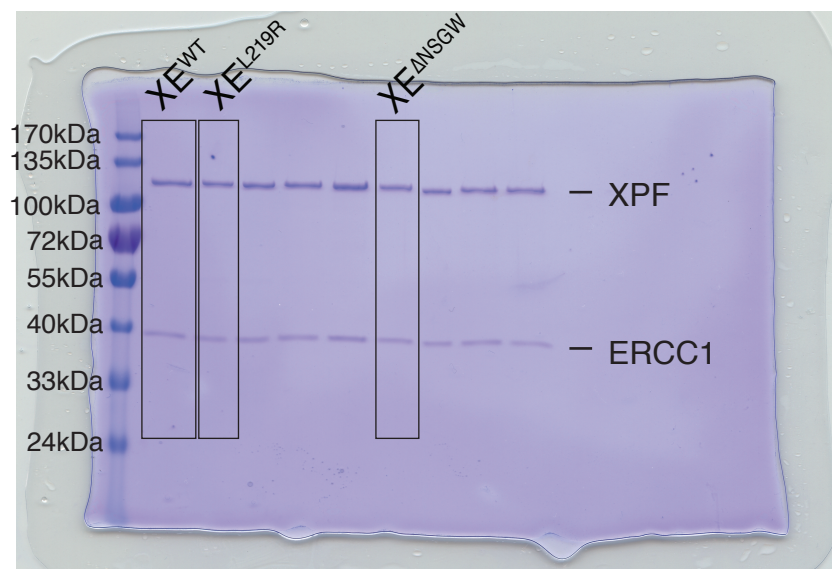

**D**

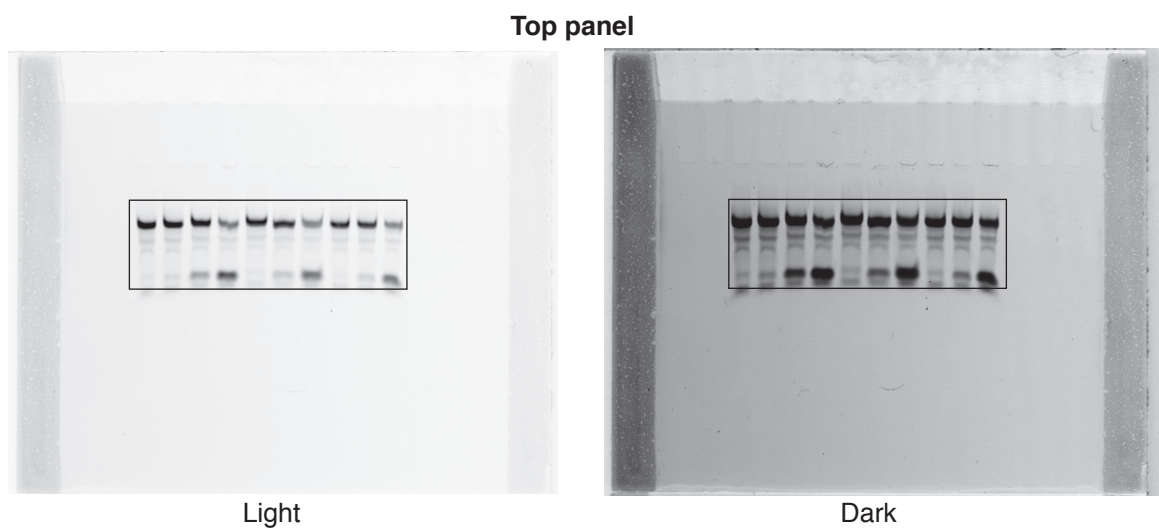

**Middle panel**

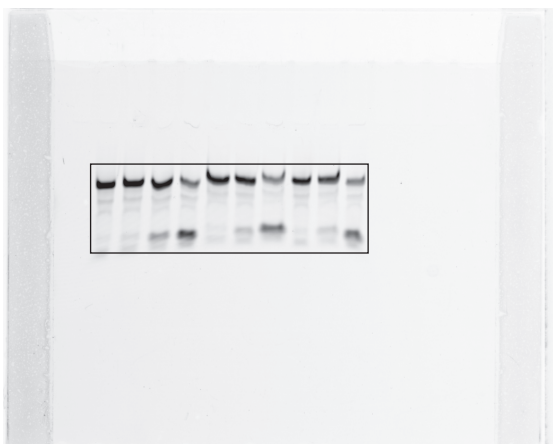

Light

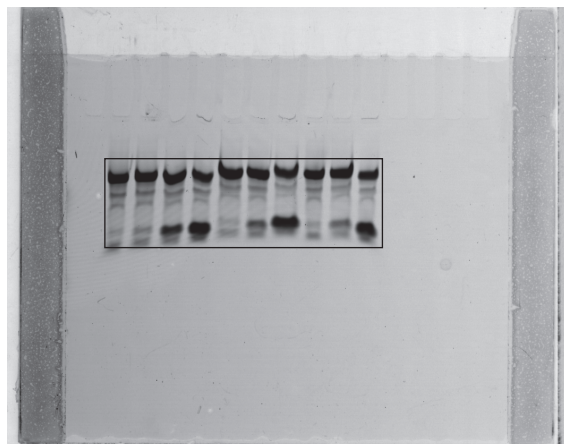

Dark

**Bottom panel**

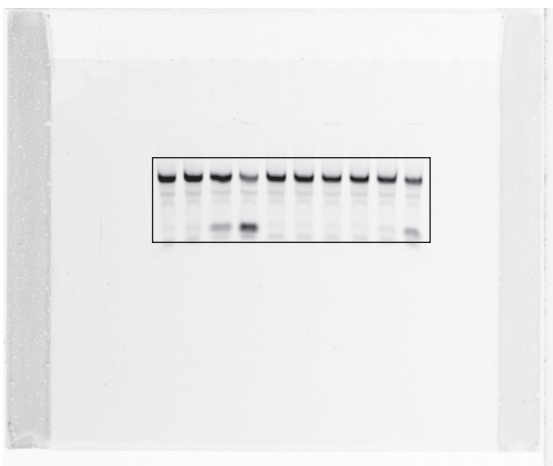

Light

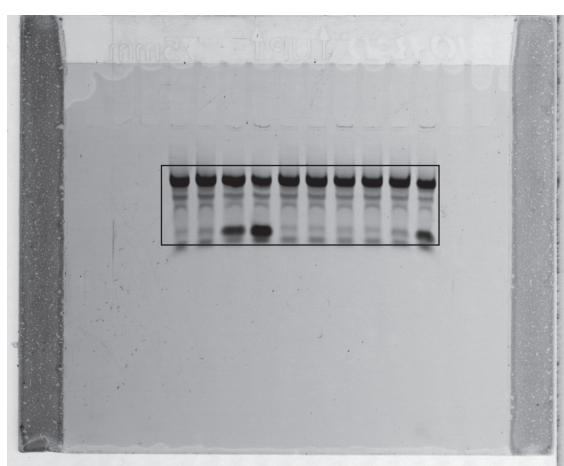

Dark

Supplement: Supplementary file 5 — Source Data for Figure 1 [file EMBJ-36-2034-s004.pdf]

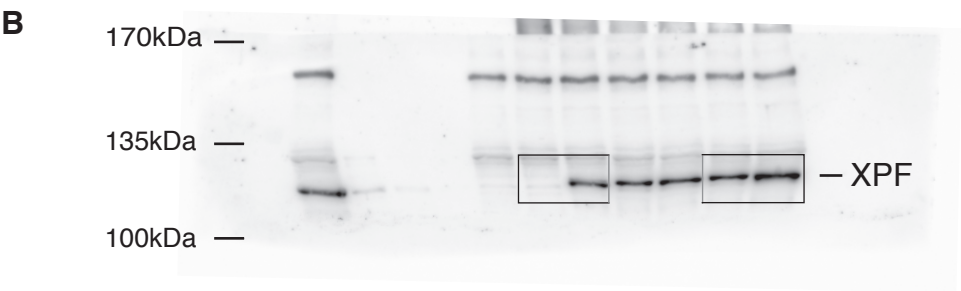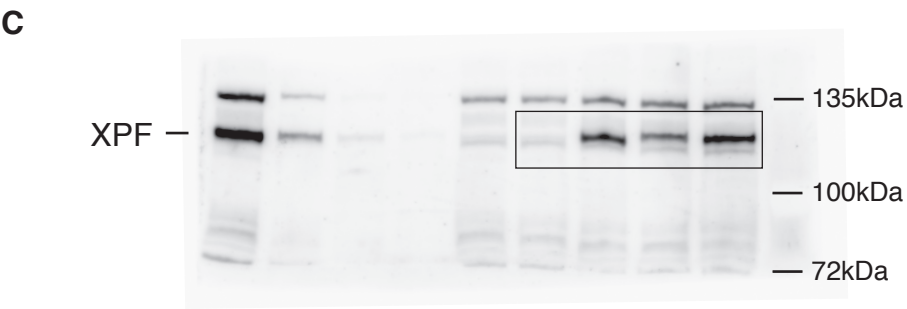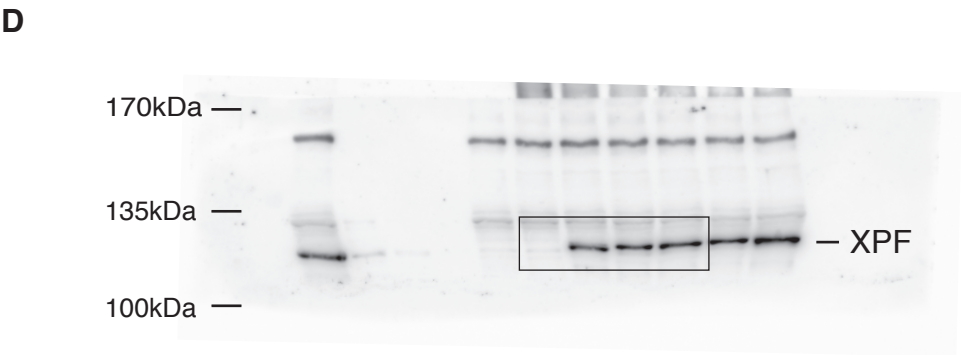

Supplement: Supplementary file 6 — Source Data for Figure 2 [file EMBJ-36-2034-s005.pdf]

**B**

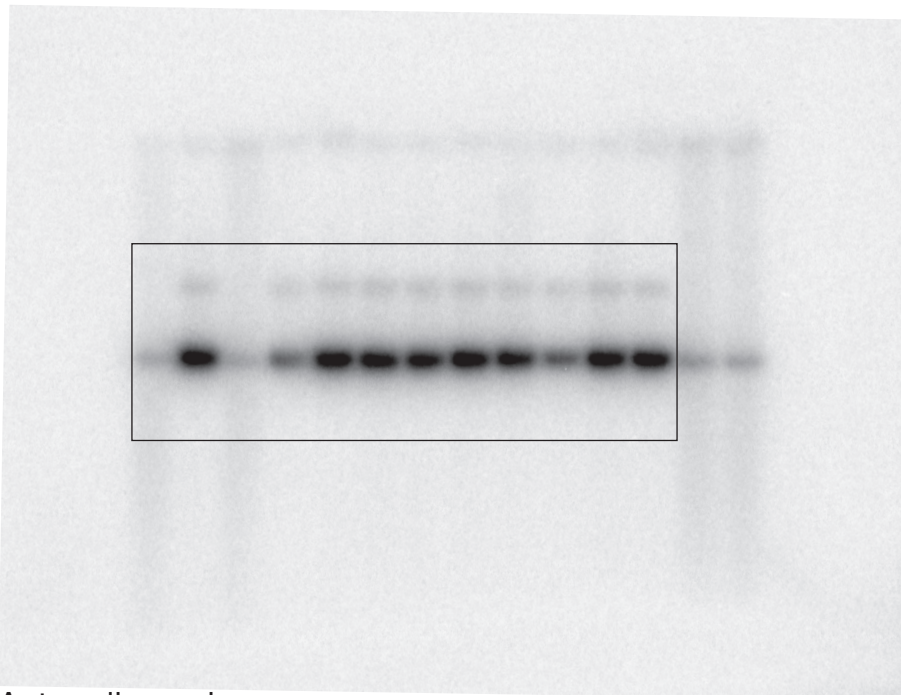

Autoradiography

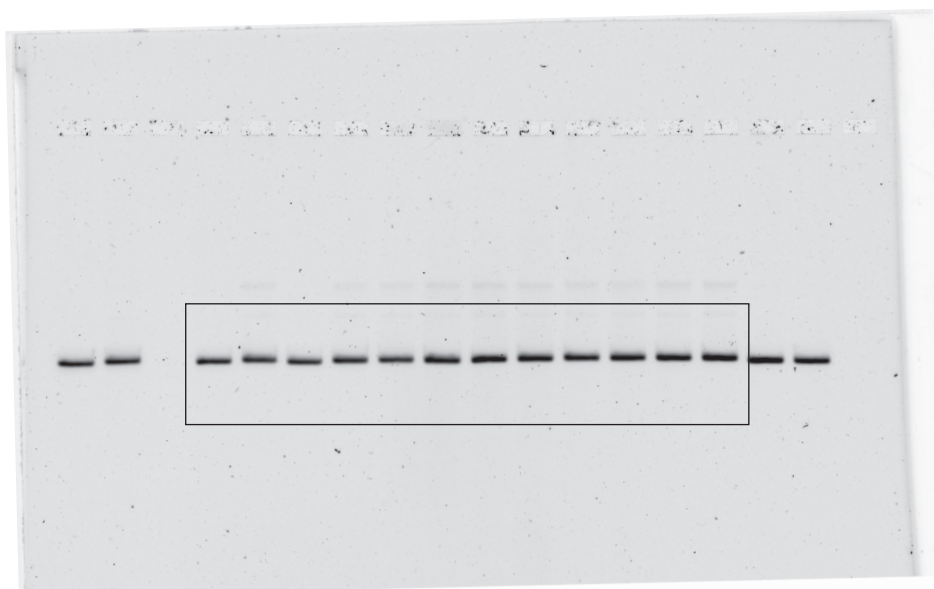

Sybr Gold

Supplement: Supplementary file 7 — Source Data for Figure 3 [file EMBJ-36-2034-s006.pdf]

Source data Fig 6

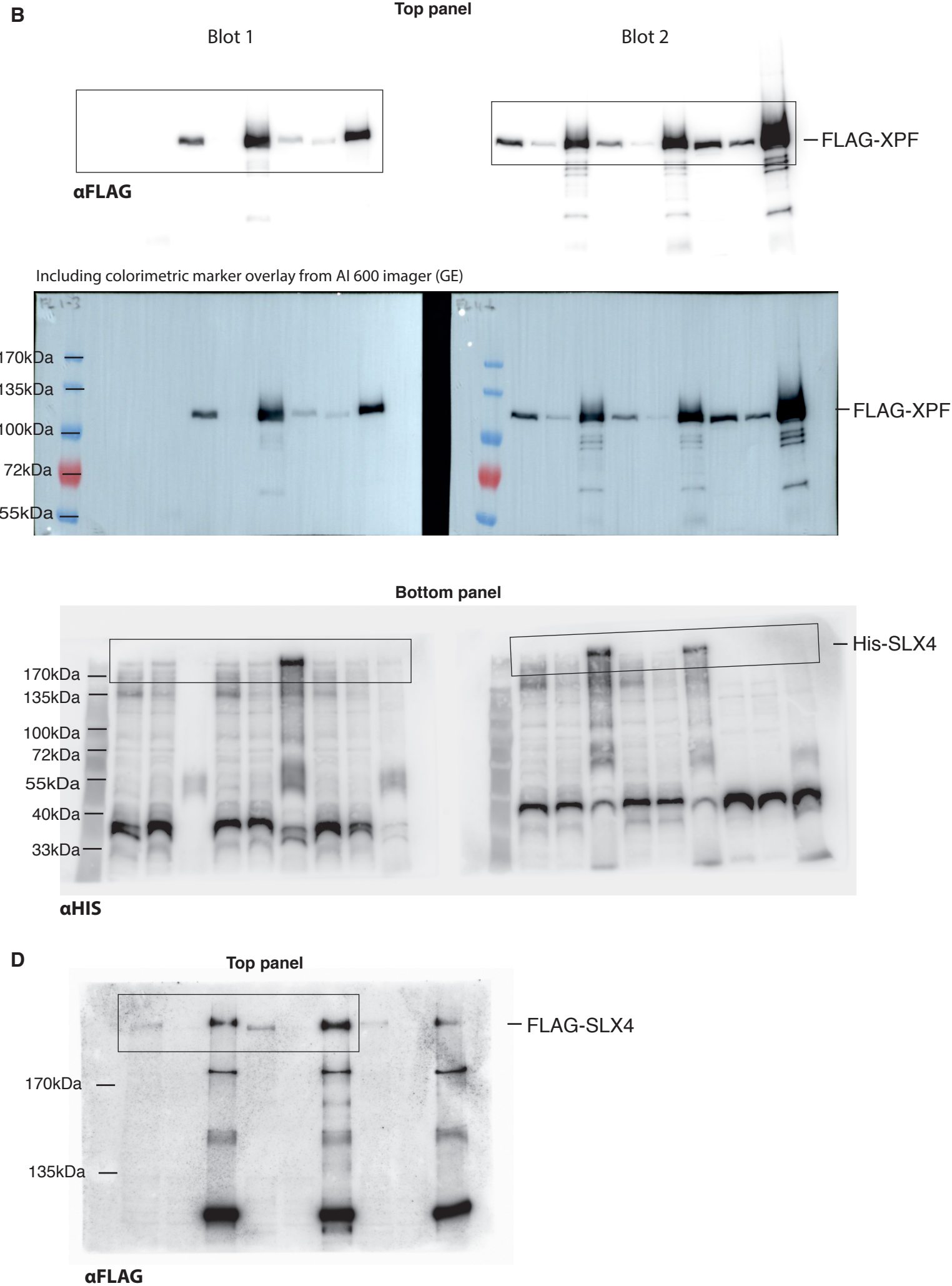

**Bottom panel**

170kDa —

135kDa —

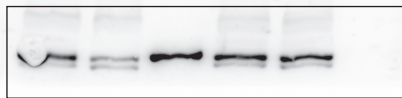

— XPF

**αXPF**

Supplement: Supplementary file 8 — Source Data for Figure 6 [file EMBJ-36-2034-s007.pdf]
